# The Transcriptome of BT-20 Breast Cancer Cells Exposed to Curcumin Analog NC2603 Reveals a Relationship between EGR3 Gene Modulation and Cell Migration Inhibition

**DOI:** 10.3390/molecules29061366

**Published:** 2024-03-19

**Authors:** Felipe Garcia Nishimura, Beatriz Borsani Sampaio, Gabrielly Oliveira do Couto, Aryane Dias da Silva, Wanessa Julia da Silva, Kamila Chagas Peronni, Adriane Feijó Evangelista, Mohammad Hossain, Jonathan R. Dimmock, Brian Bandy, Rene Oliveira Beleboni, Mozart Marins, Ana Lucia Fachin

**Affiliations:** 1 Unidade de Biotecnologia, Universidade de Ribeirão Preto (UNAERP), Ribeirao Preto 14096-900, Brazil; felipegnishi@hotmail.com (F.G.N.); beatrizborsani@gmail.com (B.B.S.); gabriellycoutossp@gmail.com (G.O.d.C.); aryanne.silva@sou.unaerp.edu.br (A.D.d.S.); wanessa.silva@sou.unaerp.edu.br (W.J.d.S.); rbeleboni@unaerp.br (R.O.B.); mmarins@unaerp.br (M.M.); 2 Instituto para Pesquisa do Cancêr (IPEC), Guarapuava 85100-000, Brazil; kcperoni@gmail.com; 3 Sergio Arouca National School of Public Health, Oswaldo Cruz Foundation, Manguinhos, Rio de Janeiro 21041-210, Brazil; adriane.feijo@gmail.com; 4 School of Sciences, Indiana University Kokomo, Kokomo, IN 46904, USA; mohoss@iu.edu; 5 College of Pharmacy and Nutrition, University of Saskatchewan (USask), Saskatoon, SK S7N 5E5, Canada; jr.dimmock@usask.ca (J.R.D.); b.bandy@usask.ca (B.B.)

**Keywords:** cancer, breast cancer, curcumin, curcumin analogs, BT-20, migration, metastasis, transcriptomics

## Abstract

Breast cancer represents a critical global health issue, accounting for a substantial portion of cancer-related deaths worldwide. Metastasis, the spread of cancer cells to distant organs, is the primary cause of approximately 90% of breast cancer-related fatalities. Despite advances in cancer treatment, conventional chemotherapeutic drugs often encounter resistance and demonstrate limited efficacy against metastasis. Natural products have emerged as promising sources for innovative cancer therapies, with curcumin being one such example. However, despite its therapeutic potential, curcumin exhibits several limitations. Analogous compounds possessing enhanced bioavailability, potency, or specificity offer a promising avenue for overcoming these challenges and demonstrate potent anti-tumor activities. Our study investigates the antimetastatic potential of the curcumin analog NC2603 in breast cancer cells, utilizing BT-20 cells known for their migratory properties. Cell viability assessments were performed using the MTT reduction method, while migration inhibition was evaluated through scratch and Transwell migration assays. Transcriptome analysis via next-generation sequencing was employed to elucidate gene modulation and compound mechanisms, with subsequent validation using RT-qPCR. The IC50 of NC2603 was determined to be 3.5 μM, indicating potent inhibition of cell viability, and it exhibited greater specificity for BT-20 cells compared with non-cancerous HaCaT cells, surpassing the efficacy of doxorubicin. Notably, NC2603 demonstrated superior inhibition of cell migration in both scratch and Transwell assays compared with curcumin. Transcriptome analysis identified 10,620 modulated genes. We validated the expression of six: EGR3, ATF3, EMP1, SOCS3, ZFP36, and GADD45B, due to their association with migration inhibition properties. We hypothesize that the curcumin analog induces EGR3 expression, which subsequently triggers the expression of ATF3, EMP1, SOCS3, ZFP36, and GADD45B. In summary, this study significantly advances our comprehension of the intricate molecular pathways involved in cancer metastasis, while also examining the mechanisms of analog NC2603 and underscoring its considerable potential as a promising candidate for adjuvant therapy.

## 1. Introduction

Breast cancer has become the most common type of cancer worldwide, occupying fourth position in terms of fatality rates [1]. The prognosis of breast cancer is influenced by factors such as the stage at diagnosis and the presence of molecular markers. However, metastasis is primarily responsible for approximately 90% of breast cancer-related deaths [2]. Likewise, treatments are also influenced by these factors. Chemotherapeutic drugs that target only a single molecular target, known as monotargeted, exhibit high specificity. However, cancer cells can develop resistance to these drugs, a fact that poses a challenge.

Since the 19th century, natural products have played a significant role in the discovery of novel compounds capable of overcoming drug resistance. Such compounds are obtained by direct isolation from natural sources, total or semi-synthesis, or the utilization of structural patterns as inspiration for the development of new molecules. Natural products serve as an important source of novel substances with significant biological activities, offering a promising alternative to the challenges encountered in the development of current chemotherapeutics [3]. Camptothecin, initially discovered in the stem wood of *Camptotheca acuminata*, stands as an example of a pentacyclic alkaloid renowned for its potent inhibition of topoisomerase I. Notably, two of its analogs, topotecan and irinotecan, have garnered approval from the Food and Drug Administration (FDA) for the treatment of various cancer types [4]. Paclitaxel presents another compelling instance, recognized as one of the most formidable anti-cancer agents available. Predominantly utilized as a first-line therapy for breast, ovarian, gastric, and non-small-cell lung cancers, its mechanism of action involves disrupting the normal progression of mitosis by inducing dysfunction in microtubule dynamics [5,6].

Curcumin, a well-known natural compound, is widely recognized for its numerous health benefits. This compound has shown therapeutic effects on different diseases, including several types of cancer, which are primarily attributed to its antioxidant and anti-inflammatory properties [7]. It also modulates various cellular signaling molecules such as proinflammatory cytokines and transcription factors like nuclear factor kappa B (NF-κB) [8].

Despite its beneficial properties, the therapeutic efficacy of curcumin is limited by its low water solubility and poor bioavailability, instability, and rapid metabolism and elimination. One approach to overcome these challenges is the use of curcumin derivatives obtained by structural modifications or synthesis of analogs [7]. Such modifications provide compounds with improved biological activities when compared with curcumin. Consequently, curcumin derivatives have shown promising potential as antitumor agents, inducing apoptosis and inhibiting the proliferation and growth of different cell lines [9].

Within its intricate structure, curcumin has seven carbon atoms intricately woven together in two phenolic rings, rendering it a subject of extensive scientific inquiry. Variations manifest in the rhizomes of Curcuma longa, where natural analogs of curcumin emerge. These analogs, characterized by a unique arrangement of five carbon atoms interlinking the phenolic rings, present a structural deviation featuring a singular ketone group. This disparity from curcumin's two ketone groups, spanning seven carbon atoms, underscores a compelling divergence warranting meticulous investigation [10,11].

The scientific community has embarked on an exploration into the contrasting attributes of five- and seven-carbon analogs. For instance, Lin et al. delved into the therapeutic potential of five-carbon analogs in combatting prostate cancer, while Robinson et al. scrutinized their efficacy in inhibiting angiogenesis, marking strides towards clarifying the therapeutic potential of these structurally distinct compounds [12,13].

In recent years, our research group, in collaboration with partners, has been dedicated to studying the antitumor activities of synthetic curcumin analogs containing the 1,5-diaryl-3-oxo-1,4-pentadienyl group in which, like the curcumin analogs cited above, five carbons intricately link the molecular rings. However, a novel addition distinguishes these analogs—a strategically placed piperidine moiety integrated into the central portion of its molecular structure. This nuanced modification holds promise for unlocking therapeutic potential in our pursuit of novel anticancer agents. Previous studies indicated that molecules containing this group possess antitumor properties attained by destabilizing the mitochondrial membrane potential and increasing the production of reactive oxygen species, leading to cytotoxicity [14]. Additionally, similar molecules have been shown in various studies to induce apoptosis and cell cycle arrest [15].

Therefore, the objective of this study was to evaluate the effects of a curcumin analog containing the 1,5-diaryl-3-oxo-1,4-pentadienyl group on the inhibition of cell migration in a breast cancer cell line. Furthermore, we aimed to investigate the mechanism of action by gene expression analysis.

## 2. Results

### 2.1. Cell Viability Suppression by Curcumin Analog NC2603

**Figure 1:**
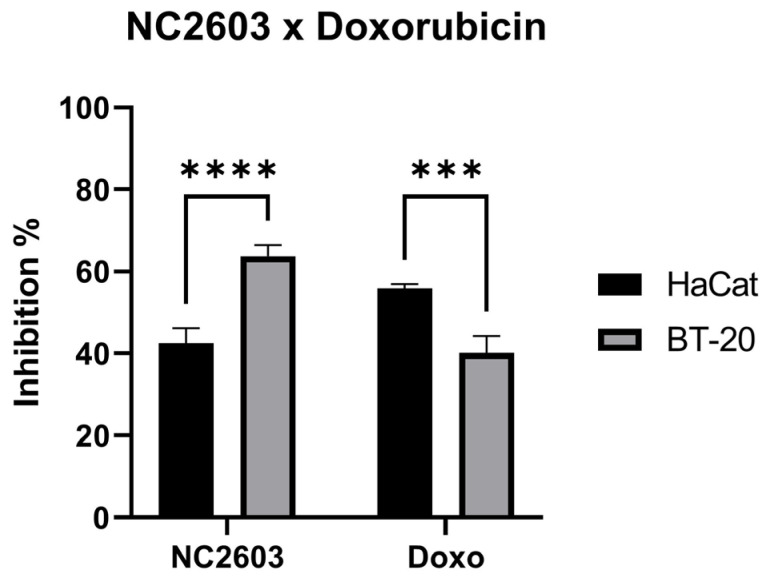
Cytotoxicity assays of NC2603 and doxorubicin, both at 5 μM, against BT-20 and HaCaT. Cells were treated for 24 h and cell viability was measured via the MTT reduction assay (*** *p* < 0.005, **** *p* < 0.001).

To assess the cytotoxic impact of NC2603, we employed the MTT reduction assay, computing cell viability inhibition values following 24 h of treatment. The cytotoxicity profiles of analog NC2603 and doxorubicin were evaluated against the BT-20 and HaCaT cell lines at an equimolar concentration of 5 μM (Figure 1). This concentration was deliberately chosen as a relatively low treatment dose to avoid inducing excessive cell death while ensuring a discernible effect. Our observations indicate that the treatments were more effective against the BT-20 cell line compared with HaCaT. Furthermore, HaCaT cells were more susceptible to the cytotoxic effects of doxorubicin treatment.

Dose–response curves were intentionally constructed to determine the IC50 of the analog. Since 5 μM induced approximately 40% cell death, a starting concentration of 20 μM was chosen, followed by subsequent halving of the concentration (20, 10, 5, 2.5, 1.25, 0.625 μM). The resulting IC50 was approximately 3.5 μM. Subsequently, this concentration was employed for treating samples from which total RNA was extracted for sequencing and subsequent validation by RT-qPCR.

### 2.2. Cell Migration Inhibition by Curcumin and Analog NC2603 at 0.75 μM

Two distinct methodologies were employed to assess the impact of treatments on cell migration (scratch and Transwell assays). First, a concentration that would not inhibit cells nor have no effect was tested via scratch assay. With the identification of the IC50 at approximately 3.5 μM, the first concentration tested was 1 μM but resulted in cellular inviability. Further experimentation at 0.5 μM exhibited no effects. Consequently, a concentration of 0.75 μM was established as optimal, striking a balance between viability and efficacy.

Given the well-documented apoptotic mechanism of doxorubicin, it was excluded from the antimetastatic assays. This strategic decision ensured a focused assessment of the specific antimigratory properties of curcumin and analog NC2603.

**Figure 2:**
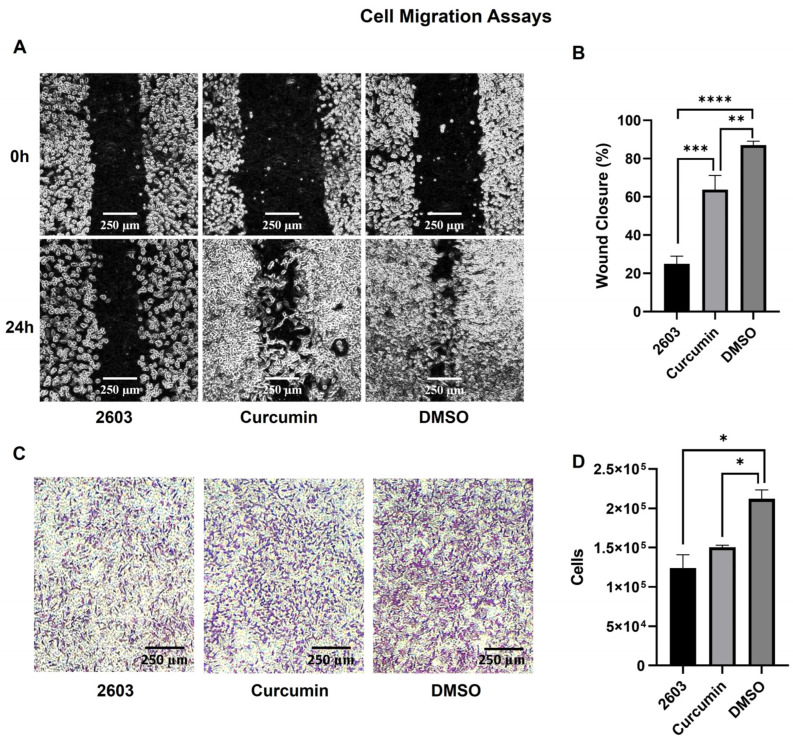
Results of the migration assays. (**A**) Comparative image of the scratch assay for NC2603 and curcumin (both at 0.75 μM) and DMSO (<0.01%) before and after 24 h of treatment (scale bar: 250 μm, 10×). (**B**) The scratch assay images were analyzed with ImageJ software to measure wound closure. The graph shows the percentage of wound closure (** *p* < 0.005, *** *p* < 0.0005, **** *p* < 0.0001). (**C**) The purple dots are cells stained with Giemsa attached to the downside of the Transwell membrane, in other words, cells that migrated (scale bar: 250 μm, 10×). (**D**) After detaching from the membrane with trypsin, the cells were counted in a Neubauer chamber. The graph shows the number of cells for each treatment (* *p* < 0.05).

Qualitatively, the two treatments inhibited cell migration in both assays. Additionally, a noticeable difference in cell morphology was observed in the migration assay. After 24 h of treatment with curcumin and DMSO, the cells returned to a more elongated shape, which was not observed in the treatment with NC2603 (Figure 2A,B).

In quantitative terms, analog NC2603 inhibited approximately 71% of wound closure in the scratch assay and approximately 41% of cell migration through the membrane in the Transwell assay. On the other hand, curcumin inhibited 27% of wound closure and 29% of migration (Figure 2C,D).

### 2.3. Differential Gene Expression Analysis: Uncovering Key Genes Regulated by Analog NC2603

**Figure 3:**
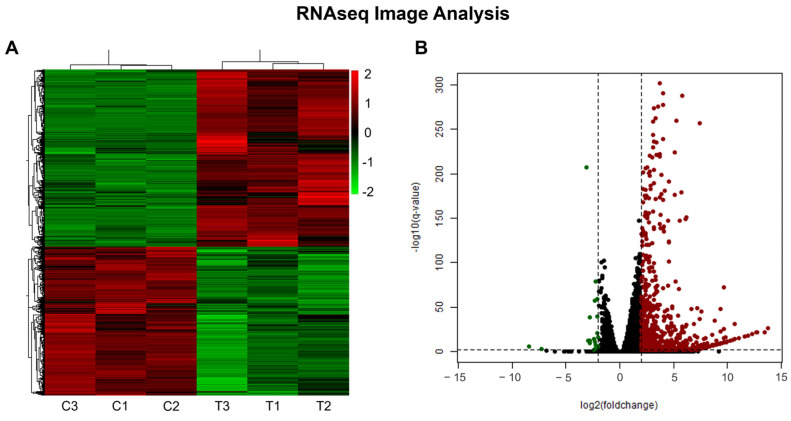
Gene expression data. (**A**) Heatmap displaying the total differential gene expression after 24 h of 10,620 genes, comparing the control (C) and treatment (T) conditions. (**B**) Volcano plot illustrating differential gene expression analysis after applying a cutoff of adjusted *p*-value (PAdj) < 0.01 and log2FoldChange ≤ −2 and ≥2. This resulted in the identification of 714 genes that exhibited significant changes, demonstrated by the green dots (repressed) and by the red dots (induced).

The analysis of differential expression based on the generated sequencing data revealed a total of 10,620 DEGs (Figure 3A) between the control and treatment with 3.5 µM of the analog NC2603 (4849 repressed and 5771 induced). To refine the results, a cutoff was applied using an adjusted *p*-value < 0.01 and log2FoldChange ≤ −2 and ≥2 as parameters, which reduced the number of DEGs to 714 (21 repressed and 693 induced) (Figure 3B). These genes were further analyzed based on their functions in the carcinogenesis process.

### 2.4. Enrichment Analysis

**Figure 4:**
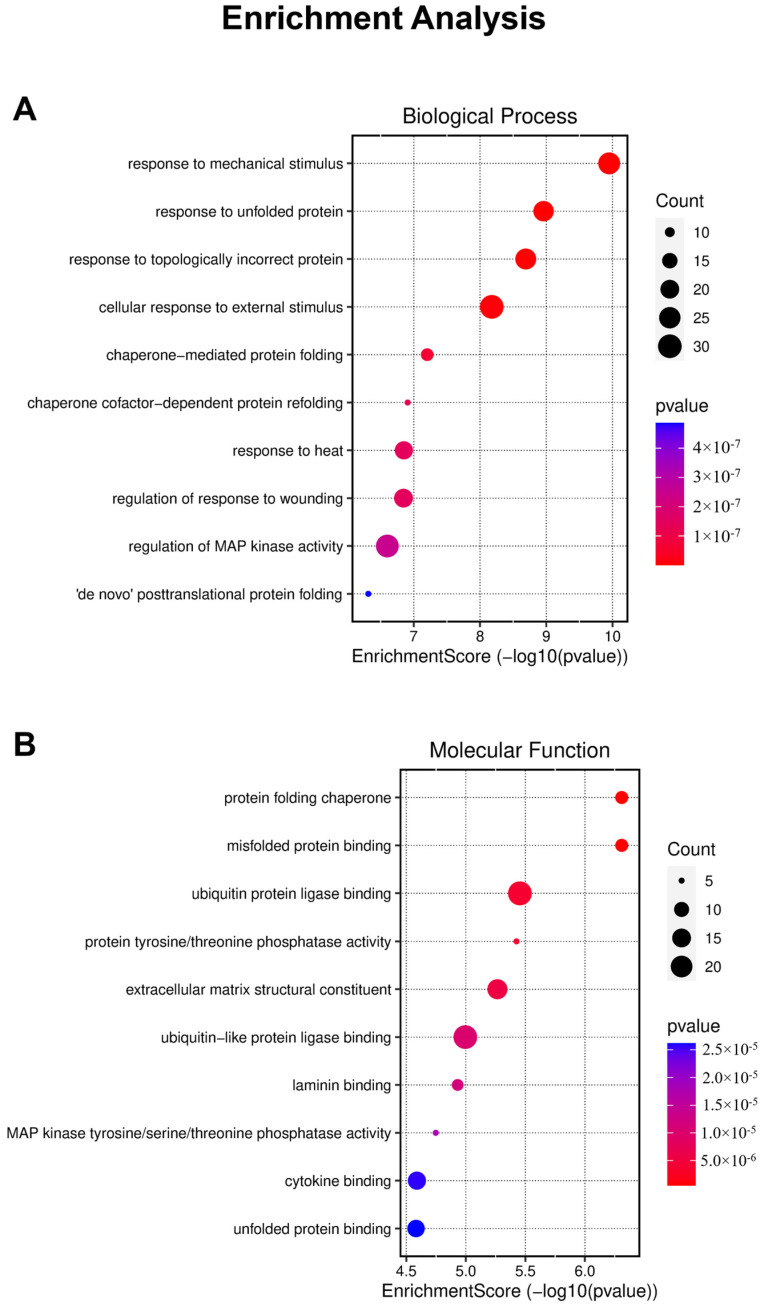
Results of enrichment analysis, providing a visual representation of the number and score of genes categorized into biological processes (**A**) and molecular functions (**B**).

This analysis enabled us to identify significantly overrepresented GO terms among the DEGs, providing valuable insights into the affected biological processes and molecular functions within our experimental system (Figure 4). However, it is noteworthy that the analysis did not provide substantial evidence regarding specific biological effects such as migration inhibition or cell death.

### 2.5. Validation of EGR3 Gene Targets: Unveiling the Migration Inhibition Mechanism

A recent investigation shed light on the intricate interplay among the expressions of six genes (EGR3, ATF3, EMP1, SOCS3, ZFP36, and GADD45B), unveiling a transcriptional activation cascade whereby EGR3 orchestrates the induction of the other genes [16]. Interestingly, our analysis of RNAseq data aimed at elucidating genes implicated in the migration process unveiled a noteworthy observation: the induction of these same genes after NC2603 treatment. As a result, acknowledging the potential significance of these genes in mediating the observed migration inhibition phenomenon, and subsequent to the absence of evidence for metastasis-related genes in the enrichment analysis, these specific genes were strategically chosen for subsequent validation.

**Figure 5:**
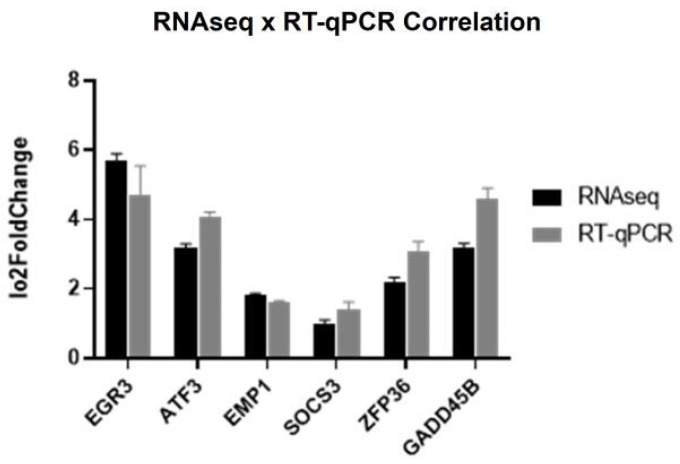
Log2FoldChange values obtained in the RNAseq and RT-qPCR validation experiments (Pearson correlation: R = 0.8462, *p* < 0.05; Spearman correlation: R = 0.9856, *p* < 0.006).

Correlation tests between the RNAseq and RT-qPCR results (Figure 5) revealed a significant correlation, confirming the accuracy and reliability of our gene expression measurements across the two techniques.

## 3. Discussion

As mentioned, curcumin has seven carbon atoms intricately woven together in two phenolic rings. Phenolic moieties harbor hydroxyl groups poised for hydrogen donation, facilitating the neutralization of free radicals. Conversely, the diketone segment boasts a central carbon nexus binding two ketone groups, each tethered to two hydrogen atoms. This structural motif engenders an inherent predisposition for hydrogen dissociation, potentiated by the dipolar moment induced by two adjacent oxygen atoms. This nuanced interplay of functional groups underscores curcumin's potent antioxidative and reactive oxygen species scavenging capabilities, as well as its reactivity in cellular processes [11].

The compound NC2603 represents a synthetic curcumin analog featuring a distinctive molecular architecture characterized by five carbon atoms bridging its phenolic moieties alongside a piperidine moiety intricately integrated into its central framework. Exhibiting a pale yellow crystalline appearance in contrast to the strong yellow color of curcumin, NC2603 manifests insolubility in aqueous environments while demonstrating solubility in dimethyl sulfoxide (DMSO). The proposed mechanisms of action for compounds such as NC2603, also called 3,5-bis(benzylidene)-4-piperidones, have been documented in the literature [17]. These mechanisms involve elevation of intracellular reactive oxygen species (ROS) levels, inhibition of oxygen consumption by the electron transport chain, and reduction of mitochondrial membrane potential. Notably, compounds like NC2603, which contain the 1,5-diaryl-3-oxo-1,4-pentadienyl group, demonstrate thiol alkylation activity.

In studies on human colon cancer cell lines, these compounds exhibited dose-dependent inhibition of cell viability and induced cell cycle arrest at the G2/M phase. Additionally, treatment effectively triggered apoptosis by augmenting intracellular ROS accumulation, reducing mitochondrial membrane potential.

Trying to determine whether these compounds are less toxic to human non-malignant cells, results indicated that these compounds displayed greater toxicity to the tumor cell line [17]. Here, we investigated the cytotoxic effects of NC2603 on both HaCaT cells, a non-malignant cell line derived from human skin keratinocytes, and BT-20 cells, a breast cancer cell line, and it also showed greater affinity to the tumor cell line. Furthermore, it also exhibited superior inhibitory effects compared with doxorubicin, which notably exerted more pronounced effects on HaCaT cells.

According to the latest World Health Organization (WHO) survey, there were approximately 2.26 million new cases of breast cancer in 2020, accounting for 12.5% of all new cancer diagnoses. In addition, breast cancer resulted in 685,000 deaths, corresponding to 7% of all cancer-related deaths [18]. Furthermore, 20–30% of breast cancer patients develop metastasis.

RECIST (response evaluation criteria in solid tumors) is a standard approach for evaluating the effects of treatments on solid tumors, primarily focusing on tumor reduction [19]. However, this criterion overlooks important factors such as the ability of anti-tumor drugs to prevent or inhibit metastasis. Moreover, adjuvant therapy is a crucial strategy for preventing recurrences and providing post-diagnostic treatment. Considering these limitations, it is important to explore drugs with antimetastatic effects as potential alternatives to adjuvant treatment.

Curcumin and its analogs have gained significant attention due to their diverse biological activities [7]. One noteworthy activity is the inhibition of cell migration. Some studies have demonstrated the inhibitory effects of curcumin on cell migration [20], while others have reported similar or even more potent effects using analogs [21]. In our study, we observed that analog NC2603 effectively inhibited cell migration at submicromolar concentrations (0.75 μM), whereas curcumin at the same concentration did not exert the same effect. The migration assays are part of a larger study aimed at characterizing the activities of this analog. Consequently, we performed transcript sequencing after treatment with analog NC2603, which allowed us to identify modulated genes and to gain insights into the mechanisms of action of the compound.

Metastasis involves a complex interplay of cellular mechanisms, with epithelial-mesenchymal transition (EMT) being a key phenotypic event during which cells lose epithelial characteristics and acquire mesenchymal properties [22]. The regulation of cellular pathways, transcription factors, and cytokines, including IL-6/STAT3, SNAIL, TWIST, and zinc finger E-box-binding (ZEB), plays a pivotal role in the metastatic process of cancer [23].

As previously noted, a recent study highlighted the suppressive role of the early growth response 3 (EGR3) transcription factor in metastasis. That study demonstrated its ability to activate specific genes—ZFP36, GADD45B, and SOCS3—in a prostate cancer cell line [18]. The authors proposed that EGR3 binds to the promoter regions of ATF3, EMP1, GADD45B, SOCS3, and ZFP36, thereby inhibiting cell migration. In our investigation, we observed the modulation of these genes after treatment with the analog NC2603.

Early growth response transcription factors are known to participate in diverse cellular processes such apoptosis, proliferation, and cell growth, owing to their highly conserved DNA-binding domains [24]. The EGR3 gene has been implicated as a tumor suppressor and prognostic marker in certain types of cancer. Notably, in estrogen receptor-positive breast cancer, EGR3 has been suggested to be crucial for the metastatic process [25]. In nasopharyngeal carcinoma and prostate cancer, the expression of this gene has been linked to the inhibition of cell migration [26]. All the other genes (ATF3, EMP1, GADD45B, SOCS3, and ZFP36), distinct from EGR3, have been independently demonstrated to function as migration inhibitors in cancer cells [27,28,29,30,31,32].

**Figure 6:**
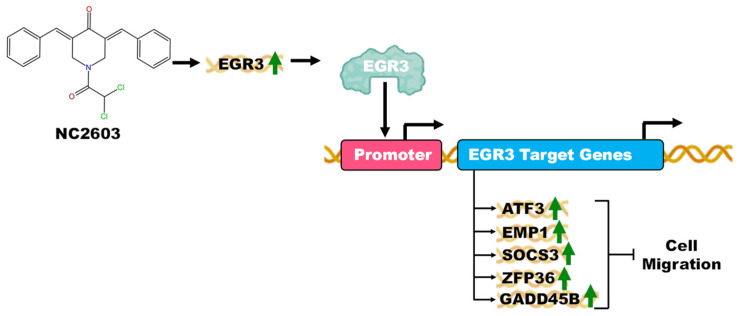
A comprehensive illustration depicting the potential mechanism of action for analog NC2603 in the inhibition of cell migration. The analog induces the expression of the EGR3 gene (green arrow demonstrating induction). The EGR3 gene, in turn, functions to promote the expression of the genes ATF3, EMP1, SOCS3, ZFP36, and GADD45B, which were also observed to be induced in gene expression assays. Each of these genes has functions in the inhibition of cell migration.

After conducting RNAseq analysis, no other significant findings regarding genes associated with metastasis were observed. Concurrently, migration assays revealed that NC2603 treatment effectively inhibited migration. Thus, we propose that the modulation of these genes may constitute the mechanism underlying the observed inhibition of cell migration (Figure 6).

In addition to these genes, the HSPA6 gene stands out as one of the genes with the highest and lowest expression levels after NC2603 treatment. This gene exhibits characteristics related to the regulation of tumorigenesis and tumor progression in different types of cancer. While the HSPA6 gene exerts inhibitory effects on these processes in certain malignancies, it seems to induce cancer progression in others [33]. Supporting this notion, in an enlightening study exploring the impact of thymoquinone treatment on a triple-negative breast cancer cell line, Shen et al. [34] found substantial upregulation of the HSPA6 gene after thymoquinone treatment. Moreover, this upregulation of HSPA6 was correlated with marked reductions in cell growth and migratory and invasive capabilities, as validated by functional assays.

## 4. Materials and Methods

### 4.1. Materials

**Figure 7:**
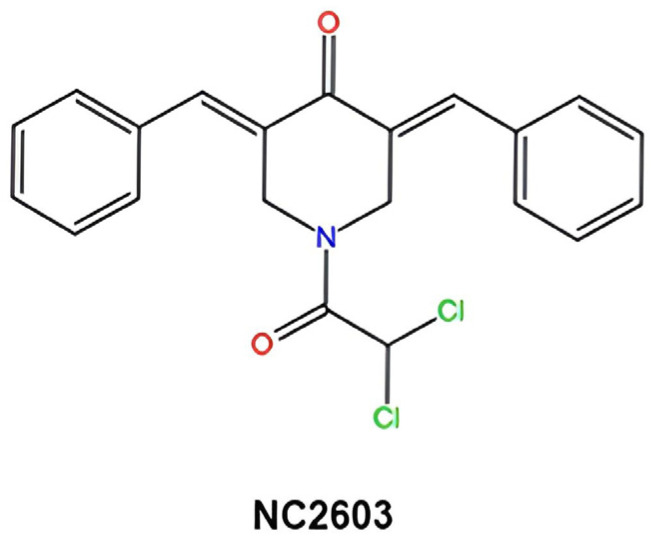
Molecular structure of curcumin analog NC2603.

The curcumin analog containing the 1,5-diaryl-3-oxo-1,4-pentadienyl group, named NC2603 (Figure 7), was synthesized by collaborators from the University of Saskatchewan, Canada [17]. Key reagents, including thiazolyl blue tetrazolium bromide (MTT), dimethyl sulfoxide (DMSO), RPMI-1640 medium, curcumin, and trypsin were purchased from Sigma-Aldrich (St. Louis, MO, USA). Sequencing was performed using Illumina technology, specifically the NovaSeq 6000 SP Reagent Kit, Illumina^®^ Stranded mRNA Prep, Ligation, and IDT^®^ for Illumina^®^ RNA UD Indexes Set A, Ligation (San Diego, CA, USA). For RT-qPCR, the SYBR^®^ Green JumpStart™ Taq ReadyMix™ and primers were also purchased from Sigma-Aldrich (St. Louis, MO, USA).

### 4.2. Cell Culture

The BT-20 (Banco de Células do Rio de Janeiro—BCRJ, Rio de Janeiro, Brazil), a triple-negative breast cancer cell line positive for migration, and HaCaT (Cell Lines Service GmbH, Eppelheim, Germany), a non-cancer keratinocyte cell line, were cultured in RPMI medium supplemented with 10% fetal bovine serum and a combination of antibiotics, including 100 U/mL penicillin, streptomycin, and kanamycin. The cell cultures were maintained in a humidified atmosphere containing 5% CO_2_ at 37 °C.

### 4.3. Cell Viability

The MTT reduction method was used to assess cell viability by measuring the for-mation of formazan crystals, following a modified version of the protocol proposed by Komoto et al. [35]. Cells were subcultured in 96-well plates at a concentration of 2 × 10^4^ cells/well. After an adherence period, the cells were treated for 24 h. After incubation, 20 μL of 5 mg/mL MTT in Hanks solution was added to each well and the plate was incubated for an additional 3 h. The supernatant was then removed and the formazan crystals were dissolved in 150 μL DMSO, with the plate being kept in an incubator for 1 h. Absorbance was measured at a wavelength of 550 nm in a micro-plate reader (MultiSkan FC, Thermo Fisher, Waltham, MA, USA). Three independent experiments were conducted to ensure the reliability and reproducibility of the results.

### 4.4. Scratch Assay

Cells were subcultured in 24-well plates at a concentration of 3 × 10^5^ cells/well until they reached 90% confluence. After the incubation period, a scratch was made on the cell monolayer with a sterile pipette tip. The cells were then treated with curcumin and analog NC2603 at a concentration of 0.75 µM, while the control group was treated with DMSO at a concentration < 0.1%. Images were captured at time 0 (T0) and after 24 h of incubation (T24). The wound areas of each replicate at T0 and T24 were analyzed using Image J software 1.53k.

### 4.5. Transwell Migration Assay

Corning^®^ 24 mm Transwell^®^ chambers with 8.0 µm pore polycarbonate membrane (Corning, New York, NY, USA) inserts were used for the cell migration assay. Each chamber was filled with 1 mL of cell suspension at a concentration of 3 × 10^5^ cells/mL in RPMI medium without fetal bovine serum. The chambers were then inserted into wells containing 2 mL of fresh RPMI medium supplemented with 10% fetal bovine serum, which served as a chemo-attractant, along with the treatments. Curcumin and analog NC2603 were used at a concentration of 0.75 µM, while the control group was treated with DMSO at a concentration < 0.1%. After 24 h of incubation, cells were counted using trypsin according to the manufacturer’s protocol.

To capture representative images of the assay, the cells were fixed in a mixture of 3.7% formaldehyde and 100% methanol, followed by staining with Giemsa. Three independent experiments were conducted.

### 4.6. Next Generation Sequencing

Total RNA was extracted from the control (containing only DMSO at a concentration < 0.2%) and treatment samples (containing analog NC2603 at the IC50 concentration) using the RNeasy Mini Kit (Qiagen, Hilden, Germany), following the manufacturer’s protocol. The integrity of the extracted RNA samples was assessed using the TapeStation system (Agilent, Santa Clara, CA, USA). Only samples with an RNA integrity value > 8 were included in the analysis. For cDNA library preparation, the High-Capacity cDNA Reverse Transcription Kit (Applied Biosystems, Foster City, CA, USA) was used. The samples were sequenced using the NovaSeq 6000 sequencing system, following the manufacturer’s protocol.

The quality of the sequencing reads was assessed using FastQC 0.11.9. Low-quality reads (Q < 28) were filtered using Trim Galore 0.6.7. The reads were aligned to the hg19 (GRCh37) reference genome 10 using STAR 2.7.9a (spliced transcripts alignment to a reference). Differentially expressed genes (DEGs) were obtained using the DESeq2 1.31.16 and EdgeR 3.38.0 software packages.

### 4.7. RT-qPCR

The selected genes (listed in Table 1) were validated using RT-qPCR. The qPCR analysis was conducted on an Mx3300 qPCR System (Stratagene, San Diego, CA, USA) using the SYBR^®^ Green JumpStart™ Taq ReadyMix™ kit. Three independent experiments were performed.

### 4.8. Enrichment Analysis

To investigate the biological processes and molecular functions associated with our DEGs, we performed gene ontology (GO) enrichment analysis using the R programming language. Furthermore, to enhance visualization of these enriched biological processes, we used an online data analysis website (http://www.bioinformatics.com.cn/, accessed on 29 June 2023). This website provides informative visual maps that aid in the interpretation and exploration of enriched functional categories.

### 4.9. Statistical Analysis

The migration assay data were submitted to analysis of variance (ordinary one-way ANOVA) followed by Tukey’s multiple comparisons test. For the viability assay, data were submitted to ordinary two-way ANOVA followed by Bonferroni’s multiple comparisons test. Two correlation tests were used to assess the correlation between the two datasets: a parametric test (Pearson correlation) and a nonparametric test (Spearman correlation).

## 5. Conclusions

In conclusion, this study focused on the investigation of the curcumin analog NC2603 as a potential inhibitor of cancer metastasis. The results demonstrated that NC2603 was superior in inhibiting cell migration compared with curcumin, even at submicromolar concentrations. Transcript sequencing showed that genes involved in key cellular processes related to metastasis are modulated by NC2603, including EGR3, ATF3, EMP1, GADD45B, SOCS3, and ZFP36. These genes have been implicated in various aspects of cancer progression, such as cell migration inhibition, tumor suppression, and the regulation of transcription factors and cytokines. The findings suggest that the mechanism of action of analog NC2603 involves the induction of these genes, which may contribute to the inhibition of cancer metastasis. Notably, EGR3 has been found to play a significant role as a tumor suppressor and prognostic marker in different types of cancer.

Limitations warrant consideration for future investigations. Firstly, incorporating a non-malignant breast cell line, such as MCF-10, could provide valuable insights into the comparative responses of malignant versus non-malignant cells to NC2603 treatment. Additionally, assessing protein expression through Western blot analysis alongside gene expression studies would offer a more comprehensive understanding of the molecular mechanisms involved. Furthermore, employing EGR3 gene deletion coupled with Western blot data could elucidate the precise mechanisms underlying the effects of both genes and the analog NC2603 on metastasis, thus enriching our comprehension of their interactions and therapeutic potential. Also, a question arises regarding whether the physicochemical properties of the aryl substituents exert influence on cytotoxic potencies. This query prompts further exploration into the structure–activity relationships of these compounds, shedding light on their potential as anticancer agents.

In summary, this study significantly advances our comprehension of the intricate molecular pathways involved in cancer metastasis, while also underscoring the considerable potential of analog NC2603 as a promising candidate for adjuvant therapy. These findings not only offer fresh insights into the conceptualization of targeted therapies aimed at metastasis prevention but also establish a solid groundwork for further exploration and innovation in this critical area of research.

## Figures and Tables

**Table 1:** Primer sequences for RT-qPCR.

Gene	Primer	
	Forward 5′-3′	Reverse 5′-3′
EGR3	GACATCGGTCTGACCAACGAG	GGCGAACTTTCCCAAGTAGGT
ATF3	GTTTGAGGATTTTGCTAACCTGAC	AGCTGCAATCTTATTTCTTTCTCGT
EMP1	GCCAATGTCTGGTTGGTTTCC	GAGGGCATCTTCACTGGCATA
SOCS3	GACCAGCGCCACTTCTTCA	CTGGATGCGCAGGTTCTTG
ZFP36	GACTGAGCTATGTCGGACCTT	GAGTTCCGTCTTGTATTTGGGG
GADD45B	ATTGCAACATGACGCTGGAAGAGC	GGATGAGCGTGAAGTGGATT
GAPDH	GACCACAGTCCATGCCATCACT	TCCACCACCCTGTTGCTGTAG

## Data Availability

The data are available on request from the corresponding author.
